# Levodopa exerts neuroprotective effects by suppressing microglial proinflammatory activation in a rat hemi-Parkinson’s disease model

**DOI:** 10.1016/j.ibneur.2025.12.001

**Published:** 2025-12-04

**Authors:** Noriyuki Miyaue, Mohammed E. Choudhury, Ikuko Takeda, Junya Tanaka, Ayane Takenaga, Haruto Yamamoto, Yuki Nishikawa, Naoki Abe, Masahiro Nagai, Tasuku Nishihara

**Affiliations:** a Department of Clinical Pharmacology and Therapeutics, Ehime University Graduate School of Medicine, Toon, Ehime 791-0295, Japan; b Department of Anesthesia and Perioperative Medicine, Ehime University Graduate School of Medicine, Toon, Ehime 791-0295, Japan; c Department of Molecular and Cellular Physiology, Ehime University Graduate School of Medicine, Toon, Ehime 791-0295, Japan; d Department of Anatomy and Molecular Cell Biology, Nagoya University Graduate School of Medicine, Nagoya, Aichi 466-8550, Japan; e Division of Multicellular Circuit Dynamics, National Institute for Physiological Sciences, Okazaki, Aichi 444-8585, Japan

**Keywords:** Dopamine, Microglia, CAMP, INOS, Behavior

## Abstract

Levodopa is a central medicine used for the treatment of Parkinson’s disease (PD) as a dopamine (DA) precursor that increases DA levels in the striatum. Microglia, resident macrophages in the brain, become activated in response to the progressive degeneration of nigral dopaminergic neurons in PD pathology, while releasing proinflammatory mediators that are harmful to dopaminergic neurons. DA has been shown to prevent proinflammatory activation of microglia. This study showed that DA decreases lipopolysaccharide-induced proinflammatory reactions and increases tissue repairing factors of microglia in cultured rat microglia. Levodopa was administered to 6-hydroxydopmaine (6-OHDA)-induced PD model rats for 7 days, and motor deficits were evaluated after a two-week withdrawal period. The levodopa-treated PD model rats showed a better motor function than the vehicle-treated rats. The administration of levodopa for 7 days led to an increase in DA levels and a suppression of microglial activation in the striatum, which was maintained, even at two weeks after withdrawal. These results suggest that levodopa may act in the PD brain, not only as a DA precursor, but also as an immunosuppressant to reduce neuroinflammation accompanied by PD pathology.

## Highlights

• Dopamine increased cAMP in microglia.
• Dopamine increased tissue expression of tissue repairing factor in microglia.
• Levodopa suppressed proinflammation of microglia in PD model brains.
• Levodopa increased the striatal dopamine level even after its withdrawal.

## Graphical Abstract

**Figure:**
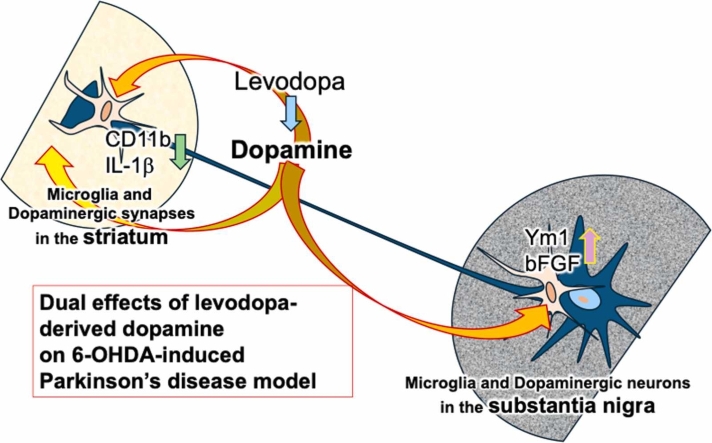

## 1 Introduction

Levodopa has long been administered to patients with Parkinson’s disease (PD) to increase dopamine (DA) levels in the striatum (Fahn et al., 2004, Holloway et al., 2000). Decreased DA levels in the striatum are the central pathology caused by the progressive degeneration of dopaminergic neurons in the substantia nigra pars compacta (SNc) (Choudhury et al., 2021a, Galvin, 2006). A decrease in the striatal DA levels causes neurological deficits, such as bradykinesia, tremor, muscle rigidity, and postural instability. These neurological deficits are ameliorated by the administration of levodopa, a blood-brain barrier (BBB)-permeable DA precursor.

Although the precise mechanisms underlying the progressive degeneration of dopaminergic neurons in the SNc remain to be elucidated, activated microglia in the SNc have been implicated in neuronal loss (Hetz and Saxena, 2017, Rocha et al., 2018). Microglia in the SNc may become secondarily activated in response to neuronal degeneration (Hirsch and Hunot, 2009, Ramirez et al., 2017; Choudhury et al., 2021a, Higaki et al., 2016). Activated microglia with a proinflammatory phenotype are thought to play aggravative roles in neurodegeneration by releasing proinflammatory mediators, such as IL-1β and TNFα (Choudhury et al., 2011), reactive oxygen species (ROS) (Abe et al., 2018, Takeda et al., 2023, Takeda et al., 2021), and nitric oxide (Ishii et al., 2015). Therefore, agents that suppress the proinflammatory activation of microglia have been shown to ameliorate the outcomes of animal models of PD (Higaki et al., 2016, Yong et al., 2004).

DA receptors are G protein-coupled ones which are expressed not only by neurons, but also by microglia (Farber et al., 2005, Nishikawa et al., 2023), and they are classified into two types: D1-like and D2-like receptors (Beaulieu and Gainetdinov, 2011). The D1-like receptors couple Gαs, thus leading to elevation of intracellular cAMP levels, and the D2-like receptors couple Gαi, leading to decreased cAMP levels (Qaddumi and Jose, 2021). Mouse and rat microglia express both D1-like and D2-like receptors (Farber et al., 2005, Nishikawa et al., 2023, Tanaka et al., 2024). The BBB-permeable selective D1-like receptor agonist SKF-81297 has been shown to suppress lipopolysaccharide (LPS)-induced proinflammatory activation in cultured microglia (Nishikawa et al., 2023). Furthermore, SKF-81297 reduced the expression of pro-inflammatory cytokines by microglia in the brains of sepsis model mice that were prepared with cecum ligation and puncture, resulting in amelioration of survival rates as well as delirium symptoms in septic mice (Tanaka et al., 2024). The notion that D1-like receptor activation leads to the suppression of microglial proinflammatory activation, suggesting the possibility that the effects of levodopa in the therapy for PD are attributable not only to elevation in striatal DA levels but also to the suppression of the neurotoxic activation of microglia. SKF-81297 has been shown to ameliorate the symptoms of 1-Methyl-4-phenyl-1,2,3,6-tetrahydropyridine (MPTP)-treated monkeys, an animal PD model (Vermeulen et al., 1993). Levodopa has also been shown to affect the microglial phenotype of MPTP-induced PD model monkeys where levodopa acts not only as a DA precursor, but also as an anti-inflammatory agent that suppresses the unfavorable actions of microglia in PD pathology (Lecours et al., 2020). Current study was aimed to investigate whether levodopa suppresses microglial proinflammatory activation in a rat hemi-PD model induced by 6-hydroxydopamine (OHDA) (Choudhury et al., 2011, Miyanishi et al., 2019). The ameliorative effects of levodopa on 6-OHDA-induced motor deficits were sustained, even at 2 weeks after levodopa withdrawal. Furthermore, the elevation of striatal DA levels was still observed at that time point, suggesting that the administration of levodopa not only provides symptomatic relief, but also treats the cause of the symptoms. Our study provides first translational potential and unique insights into the authors' primary research question regarding the neuroimmunomodulatory phenomenon of levodopa, using cell culture and animals model of Parkinson’s disease.

## 2 Materials and methods

### 2.1 Animals

This animal model study was approved by the Ethics Committee for Animal Experimentation of Ehime University, Matsuyama, Japan (approval number # 05HO1–2), and all efforts were made to minimize animal suffering and reduce the number of animals used. Home-bred 7- to 8-week-old Male Wistar rats (Clea Japan, Tokyo, Japan) were used in this study. A 6-OHDA-induced hemi-PD rat model was established as described elsewhere (Miyanishi et al., 2019). 6-OHDA (Toronto Research Chemicals Inc., Ontario, Canada) was dissolved (5 mg/500 μl) in 1 % ascorbate (Fujifilm Wako, Osaka, Japan)-containing saline (Otsuka Pharmaceutical, Tokyo, Japan). Under deep anesthesia with isoflurane, the rat head was secured in a stereotaxic apparatus (Narishige, Tokyo, Japan) and 2 μl of the 6-ODHA solution was infused at 0.5 μl/min using a 25 μl Hamilton syringe, with a 26-gauge needle into the brain parenchyma in the vicinity of the right median forebrain bundle. The needle was left after the injection for another 5 min and then slowly withdrawn. The skin on the head was sutured using surgical needle-equipped sutures (Alfresa Pharma Corporation, Osaka, Japan). On the day after 6-OHDA infusion, the rats were subcutaneously treated with either levodopa (25 mg/kg, DOPASTON, OHARA Pharmaceutical Co., Ltd., Kanagawa, Japan) containing benserazide (a DOPA decarboxylase inhibitor; 6.25 mg/kg, Wako) or vehicle (saline) twice daily for 7 consecutive days.

### 2.2 Cell culture

Primary cultures of rat microglia were prepared as reported previously (Tanaka et al., 1999). Briefly, whole forebrains from neonatal rats were removed and dissociated into individual cells using a nylon bag with 160 µm pores. Dissociated cells were cultured as mixed glial cultures in 75 cm^2^ flasks with 10 % fetal calf serum (FCS)-supplemented Dulbecco’s modified Eagle’s medium (DMEM; Fujifilm Wako). Eleven days later, microglial cells were obtained from the cultures by agitating the flasks at 200 rpm for 1 h. LPS from *Escherichia coli* serotype 055:B5 (Sigma-Aldrich, Burlington, MA) was added at a concentration of 1 μg/ml to the culture medium, E2 medium (DMEM containing 10 mM HEPES [pH 7.3; Gibco, Grand Island, NY], 4.5 mg/ml of glucose, 5 μg/ml insulin, 5 nM sodium selenite, 5 μg/ml transferrin [Gibco] and 0.1 mg/ml bovine serum albumin [Sigma-Aldrich]) to activate microglial cells for 6 or 24 h. Both activated or non-activated cells were treated with or without DA (10 μM, LKT Laboratories Inc., St Paul, MN).

### 2.3 Enzyme-linked immunosorbent assay (ELISA)

To evaluate the cAMP response, primary rat microglial cells were cultured with LPS for 3 h and then incubated in LPS medium containing 100 μM of the PDE IV inhibitor rolipram (Tocris Bioscience, Bristol, UK) for an additional 20 min (Nishikawa et al., 2023). The medium was changed to LPS medium containing 50 μM rolipam with or without 10 μM DA and incubated for another 20 min. After 20 min of incubation, lysates prepared from microglial cells were subjected to cAMP determination using an ELISA kit (Cayman Chemical, Ann Arbor, MI, USA) according to the manufacturer’s protocol. To evaluate the iNOS protein expression, primary microglial cells were incubated for 24 h in lipopolysaccharide (LPS) medium with or without DA. After incubation, cell lysates were prepared with RIPA buffer (Fujifilm Wako) and iNOS content was quantified using an iNOS ELISA kit (Cusabio, Houston, TX, USA) according to the manufacturer’s protocol. The samples were measured in duplicate and the readings from each sample were normalized to the protein concentration.

### 2.4 Immunoblotting

The ventral midbrain tissues from the ipsilateral side were immediately homogenized in 10 volumes of Laemmli’s sample solution, which contained 3 % sodium dodecyl sulfate (SDS). The lysates were then further diluted with Laemmli’s sample solution and electrophoresed. The resulting electrophoresis products were transferred to PVDF membranes. Coomassie Brilliant Blue (CBB, Nacalai tesque, Kyoto, Japan) staining was employed to ascertain the equal protein content in the loading samples (Supplementary Figure). The membrane was immunoblotted with antibodies to β-actin (ACTB, mouse monoclonal, Catalog number: 281–98721, Fujifilm Wako) and tyrosine hydroxylase (TH, sheep polyclonal, Catalog number: NB300–110, Novus Biologicals, Centennial, CO, USA). The appropriate HRP-conjugated secondary antibodies (Donkey IgG; R&D Systems, Inc. Minneapolis, MN, USA) were used for visualization. The immunoreaction was detected using ECL Western Blotting Substrate (Promega, Madison, WI, USA). Immunoreactive signals were obtained with a short exposure time for ACTB and a long exposure time for TH. (Supplementary Figure). The immunoreactive bands analyzed by densitometry using ImageJ 1.43 u (Wayne Rasband, National Institute of Health, Bethesda, MD, USA). Densitometric analyses were performed as described previously (Aono et al., 2017, Choudhury et al., 2020, Higaki et al., 2016, Islam et al., 2018).

### 2.5 Immunofluorescence histochemistry

The rats were perfused with PBS for 1 min, followed by 4 % paraformaldehyde (Fujifilm Wako) for 10 min. The frontal cortex was coronally cryosectioned to a thickness of 10 μm. Primary antibodies against Iba1 (rabbit polyclonal, Product number: 012–28521, Fujifilm Wako), CD68 (mouse monoclonal ED1, Catalog number: ab31630, Abcam, Cambridge, UK), and TH (sheep polyclonal, Catalog number: NB300–110, Novus Biologicals) were used for immunohistochemical staining. After incubation with primary antibodies, sections were incubated with DyLight 488, DyLight 549, and/or DyLight 649-labeled secondary antibodies (Jackson ImmunoResearch Laboratories, West Grove, PA, USA). Hoechst 33342 (Sigma-Aldrich) was used for nuclear staining. Imaging was performed using a TiE-A1R Apo, NA 1.45, Nikon), as described elsewhere (Takeda et al., 2022).

### 2.6 Quantitative real-time RT-PCR (qPCR)

Total RNA from cells or brain tissues was isolated using the Maxwell RSC simplyRNA Tissue/Cells Kit (Promega). Approximately 500 ng of total RNA was used and cDNA was synthesized using ReverTra Ace qPCR RT Master Mix with a gDNA remover kit (Toyobo, Osaka, Japan). The cDNA samples were diluted 3-fold, and qPCR was performed in triplicate using an MJ mini instrument (BioRad, Hercules, CA, USA) using THUNDERBIRD™ Next SYBR® qPCR Mix (Toyobo). Glyceraldehyde-3-phosphate dehydrogenase (GAPDH) was used as the reference gene to normalize the gene expression. All PCR primer sequences are listed in Supplementary Table 1. The qPCR data are shown as the percentage of the GAPDH mRNA expression level, which was calculated as 100 × 1/2^(Ct of target gene –Ct of GAPDH gene)^ (Islam et al., 2018).

### 2.7 High-performance liquid chromatography

The DA content in the right striatum was determined using HPLC. Tissues were homogenized in 0.2 M perchloric acid containing 5 mM ethylenediaminetetraacetic acid and 3,4-dihydroxybenzamine (Fujifilm Wako with an ultrasonic cell disruption device. The lysate was then incubated on ice for 30 min and centrifuged at 20,000 × *g* at 4°C for 10 min. The supernatant was filtered and aliquots were injected into an HPLC system with a reversed-phase column (Shimadzu Corporation, Kyoto, Japan). Synthetic DA (Fujifilm Wako) was used as a standard (Miyanishi et al., 2019).

### 2.8 Flow cytometry (FCM)

The striatal tissues were dissociated into single cells using an adult brain dissociation kit following the manufacturer’s instructions (Miltenyi Biotec, Bergisch Gladbach, Germany), and the dissociated cells were processed as previously described (Abe et al., 2018). Zombie Violet (BD Biosciences) treatment was performed to eliminate dead cells. The samples were then incubated on ice with anti-mouse CD32 antibody (BD Biosciences, 1:100) to block Fc receptors. The cells were incubated with fluorescence- labeled antibodies (Pacific Blue™ anti-rat CD45 antibody and Alexa Fluor® 647 anti-rat CD11b/c antibody, BioLegend, San Diego, CA, USA) on ice for 30 min. After centrifugation, the pellets were resuspended in 250 ml of PBS containing2 % fetal bovine serum. Flow cytometry analyses were performed on a Gallios flow cytometer (Beckman Coulter, Tokyo, Japan) using gating strategies as described previously (Nishikawa et al., 2023). Data were analyzed using FlowJo (version.7.6.5, Treestar, Ashland, OR, USA).

### 2.9 Measurement of the oxygen consumption rate (OCR)

Rat adult microglia were isolated using CD11b/c (Microglia) MicroBeads (Miltenyi Biotec) and plated in E2 medium in Seahorse 8 well cell culture plates (Agilent Technologies, Santa Clara, CA, USA) at a concentration (5 × 10^4^ cells/well) with or without DA (10 μM) for overnight incubation. OCR was measured using a Seahorse XFp Extracellular Flux Analyzer and Seahorse Cell Mito Stress Test kit, as described elsewhere (Abe et al., 2018).

### 2.10 Assessment of motor disturbance

**(A) Cylinder test**

The cylinder test was used to assess motor deficits (Miyanishi et al., 2019) with slight modifications. The rat was placed in a cylinder, and the forelimb was used to explore the walls. The experimenter counted the number of wall contacts by each forelimb for 5 m. The cylinder test score was calculated as the percentage of touches (contralateral side/ipsilateral side).

**(B) Forepaw adjustment steps test (FAS)**

One day after, the FAS test was used to assess the motor deficit in the left forelimb (Miyanishi et al., 2019) where hindlimbs and one forelimb were restrained so that only one forelimb was forced to support the body weight on a moving (9 cm/s) treadmill (Animal treadmill Exer 3/6, Columbus Instruments, Columbus, OH). The rats were then moved laterally across a treadmill. The test was performed three times for 20 s each time. ‘FAS score’ was percentages obtained by dividing the number of steps by the contralateral forepaw with that by the ipsilateral one. Lower scores indicate greater motor impairment. Measurements were performed in triplicate.

### 2.11 Statistical analysis

Data are expressed as the mean ± SD. Statistical data were analyzed using a two-tailed Mann-Whitney test, two-tailed paired *t*-test, or a one- or two-way analysis of variance (ANOVA) with Tukey’s post hoc test (the method used in each experiment is described in the figure legends). All analyses were performed using Prism 9 (GraphPad Software, La Jolla, CA, USA). P values of < 0.05 were considered significant in all tests. The single, double, triple, and quadruple asterisks in the graphs indicate statistical significance at P < 0.05, 0.01, 0.001, and 0.0001, respectively.

## 3 Results

### 3.1 Effects of DA on rat primary cultured microglia

**Fig. 1:**
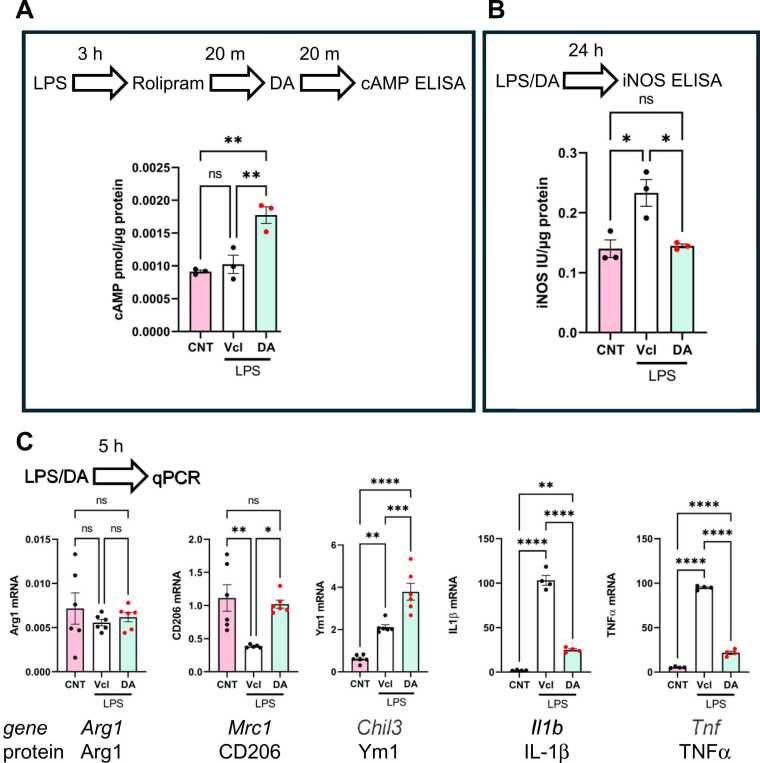
Effects of dopamine (DA) on rat primary cultured microglia.DA increased intracellular cAMP levels in microglia in the presence of LPS. *n* = 3. (A); The iNOS protein expression as evaluated by an ELISA. DA abolished the LPS-induced elevation of the iNOS expression. *n* = 3 (B). The expression of Arg1, CD206, Ym1, IL-1 β, and TNFα at mRNA levels were evaluated by qPCR. In the presence of LPS, DA increased the expression of CD206 and Ym1 but not Arg1 (*n* = 6), (C). The expression of IL-1β and TNFα mRNA was suppressed by DA (*n* = 4). CNT: (normal control, media only), Vcl: LPS containing medium; DA: LPS and dopamine containing medium. A one-way ANOVA and Tukey’s multiple comparison test. *, **, ***, **** indicate statistical significance vs. at p < 0.05, 0.01, 0.001, and 0.0001, respectively.

The expression of DA receptors (DRs) in rat primary cultured microglia has been described elsewhere (Nishikawa et al., 2023). To confirm that D1-like receptors function in the presence of LPS, microglial cells were incubated with LPS, with or without DA, and then subjected to intracellular cAMP determination using an ELISA kit. To prevent cAMP degradation by phosphodiesterase (PDE), cells were incubated with rolipram, an inhibitor of cAMP-specific PDE (PDE IV), prior to incubation with DA or vehicle (Vcl), as shown in Fig. 1A. DA elevated cAMP levels in LPS-treated microglial cells. DA suppressed the expression of LPS-induced NO synthase (iNOS) protein (Fig. 1B). DA increased the expression of genes encoding the alternatively activated microglial cell markers CD206 and Ym1, but not Arg1 (Jurga et al., 2020) in LPS-treated cells (Fig. 1C). DA decreased the expression of the proinflammatory cytokines IL-1β and TNFα. Thus, it is suggested that DA inhibits proinflammatory activation, but enhances the alternative activation of microglia *in vitro*.

### 3.2 Sustained ameliorative effects of levodopa even after its withdrawal

**Fig. 2:**
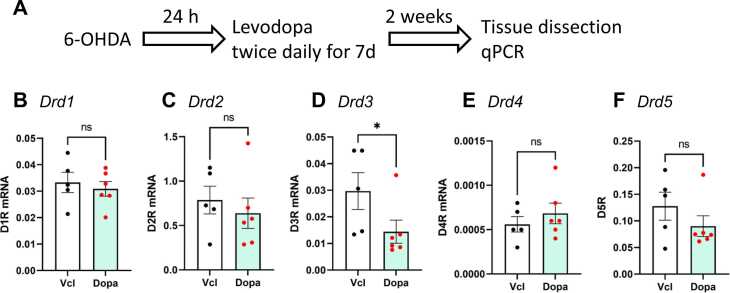
Expression of DA receptor genes in the ventral midbrain. Experimental schedule (A). The expression of genes encoding DA receptors *DRD1*, *DRD2*, *DRD3*, *DRD4*, and *DRD5* in the right ventral midbrain of 6-OHDA-treated rats with or without levodopa administration (B-F). The expression levels of all 5 genes were determined. Levodopa (shown as Dopa in the graphs) administration reduced the expression of *DRD3* (C) but not other DA receptors. *n* = 5 (Vcl) and *n* = 6 (Dopa). * indicates statistical significance versus Vcl at P < 0.05.

As shown in Fig. 2, the mRNA expression of 5 DRs in the ipsilateral ventral midbrain tissues was detected by quantitative RT-PCR (qPCR) 3 weeks after 6-OHDA injection. The administration of levodopa twice per day for a week did not affect the expression of DRDs, with the exception of DRD3.

**Fig. 3:**
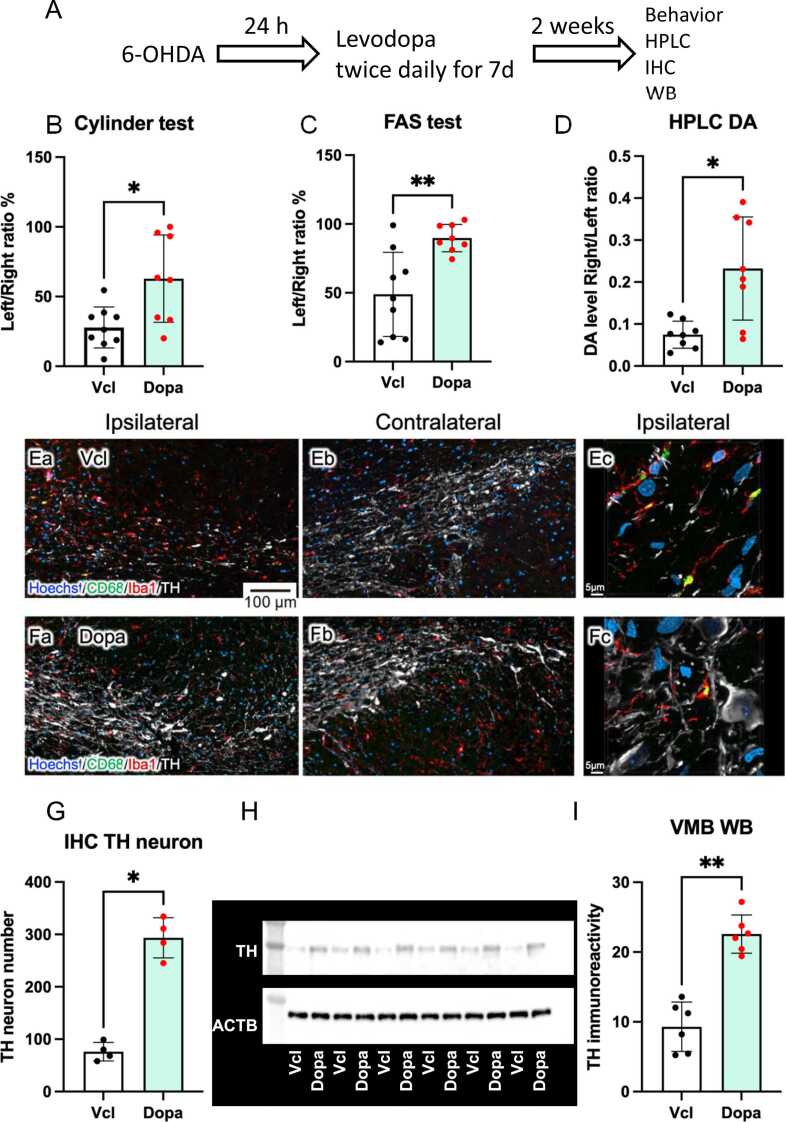
Ameliorative effects of levodopa on PD model rats. Experimental schedule (A). Cylinder (B), and FAS (C) tests showed that the administration of levodopa ameliorated motor deficits of the left forelimb. n = 9 (Vcl), n = 8 (levodopa; denoted as Dopa in the graphs). As revealed by HPLC, the striatal DA levels shown as the right/left ratio were increased, even at two weeks after levodopa withdrawal (D). Representative immunohistochemically stained images of dopaminergic neurons in the SNc of vehicle (*E*)- and levodopa (F)-treated rats. The number of TH^+^ dopaminergic neurons in the SNc were counted. n = 4 (G). Immunoblot showing TH and ACTB immunoreactivity in the ventral midbrain of vehicle- and levodopa-treated rats. (H). Densitometric analyses of immunoblot data (I) (n = 6), Please refer to the supplementary figure for the original image of immunoblot, which depicts membranes and gels. Data are expressed as the mean ± SD. Two-tailed Mann-Whitney test. *, ** indicate statistical significance vs. Vcl at p < 0.05 and 0.01, respectively.

If DA derived from levodopa has an immunosuppressive effect on activated microglia, preventing dopaminergic neuronal degeneration, its ameliorative effects on PD-induced neurochemical changes and motor dysfunctions should be sustained even after the withdrawal of levodopa administration. Therefore, we administered levodopa intraperitoneally twice daily for 7 days, starting 24 h after 6-OHDA treatment. Two weeks after the last administration, the PD model rats were subjected to cylinder and FAS tests, and the brains were dissected for qPCR and determination of the DA levels in the striatum. Cylinder and FAS tests are better behavioral tests to predict striatal DA levels in the 6-OHDA-induced hemi-PD model rats than apomorphine, rotarod, beam, and open field tests, as described elsewhere (Miyanishi et al., 2019). Even after the two-week withdrawal, the motor functions of levodopa-treated rats were better than those of vehicle-treated rats (Figs. 3B and 3C). The striatal DA level was higher in levodopa-treated rats than in vehicle-treated rats (Fig. 3D). Immunohistochemical staining showed more tyrosine hydroxylase-expressing (TH^+^) dopaminergic neurons in the SNc of levodopa-treated rats than in vehicle-treated rats (Figs. 3F, 3G, 3H). Immunoblot revealed that more TH protein was present in the right ventral midbrain of levodopa-treated rats than in vehicle-treated rats (Fig. 3I).

**Fig. 4:**
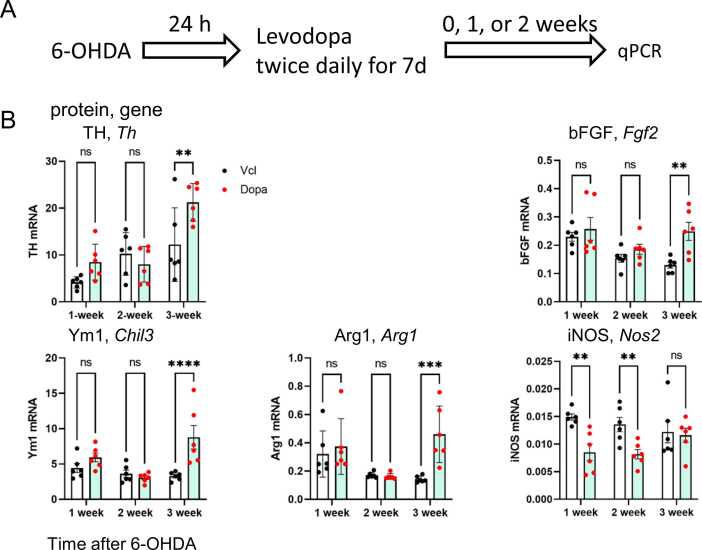
Kinetic changes in the gene expression levels in the right ventral midbrain. The expression of genes encoding TH, bFGF, Ym1, Arg1, and iNOS were evaluated by qPCR. Total RNA was collected from the right midbrain 1, 2, and 3 weeks after 6-OHDA treatment. *n* = 6. Data are expressed as the mean ± SEM. Two-way ANOVA and Tukey’s multiple comparison test. *, **, ***, **** indicate statistical significance vs. Vcl groups at p < 0.05, 0.01, 0.001, and 0.0001, respectively.

qPCR experiments were performed to determine the kinetic changes in gene expression in the ipsilateral (right) ventral midbrain containing the SNc (Fig. 4). The gene expression of TH, an essential enzyme for DA synthesis, increased in levodopa-treated rats 3 weeks after 6-OHDA treatment (or two weeks after drug withdrawal). The gene expression of alternative activation markers Arg1 and Ym1, as well as the neurotrophic factor bFGF, was elevated in the levodopa-treated rats at the 3-week time point. Furthermore, the expression of the pro-inflammatory marker iNOS was found to be inhibited by levodopa at weeks 1 and 2. However, no significant changes in iNOS expression levels were observed at 3-week time point.

### 3.3 Immunosuppressive effects of levodopa on the striatal microglia

**Fig. 5:**
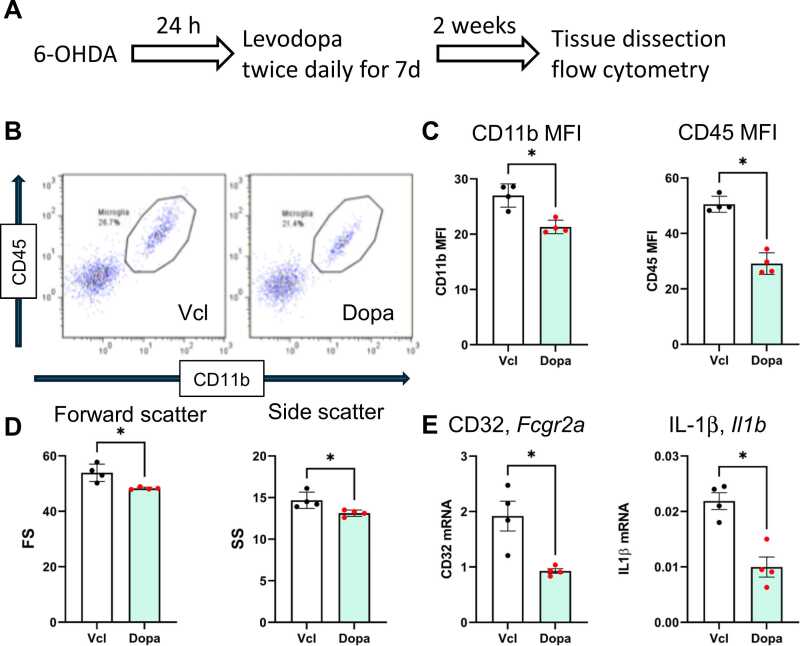
Levodopa suppressed the activation of microglia in the striatum 3 weeks after 6-OHDA treatment.Microglia in the striatum were analyzed by flow cytometry using antibodies to CD11b and CD45. Representative plots of cells from vehicle and levodopa-treated rat striata (B). The mean fluorescence intensity (MFI) of CD11b and CD45 of microglia from vehicle- and levodopa-treated striata (C). Forward and side scatters (FS and SS) of the microglia (D). cDNA was prepared from the microglia sorted from the striata. The expression of genes encoding CD32 and IL-1β was evaluated by qPCR (E). Data are shown as the mean ± SD. Unpaired *t*-test. *n* = 4. * indicates statistical significance vs. Vcl at p < 0.05.

PD pathology causes degeneration of dopaminergic axons distributed in the striatum, in addition to somata degeneration in the SNc. Degenerated axons cause the proinflammatory activation of microglia in the striatum (Ayerra et al., 2024, Chung et al., 2009). In fact, the CD11b and CD45 expression levels in the ipsilateral striatal microglia were increased relative to the contralateral microglia, as revealed by a FACS analysis 3 weeks after 6-OHDA treatment (Fig. 5B). Levodopa decreased the CD11b and CD45 expression and forward scatter (FS) and side scatter (SS) values of striatal microglia at the same time point after 6-OHDA treatment (Fig. 5C). The results showed that levodopa suppressed the proinflammatory activation of microglia in PD.

Metabolic activity is intimately correlated with proinflammatory reactions of microglia, including ROS generation. However, the flux analysis showed that DA did not significantly impact metabolic activity.

### 3.4 Effects of DA on energy metabolism in rat primary microglia

**Fig. 6:**
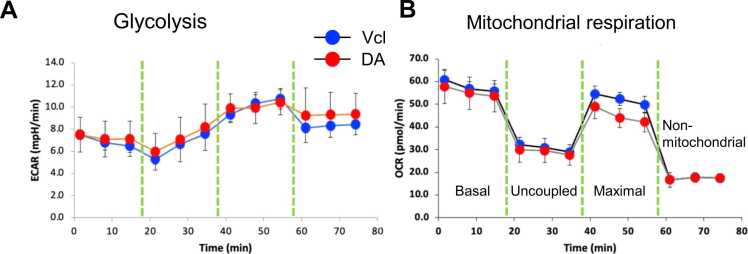
Effects of DA on energy metabolism in rat primary microglia as assessed by oxygen consumption rate (OCR) and extracellular acidification rate (ECAR).Metabolic rates of sorted striatal microglia were evaluated with a flux analyzer. The extracellular acidification rate (ECAR) reflecting glycolysis activity(A). The oxygen consumption rate (OCR) was used to evaluate mitochondrial oxidative phosphorylation. DA did not affect apparently the metabolic rates of the striatal microglia (B). Unpaired *t*-test. *n* = 3. * indicates statistical significance vs. Vcl at p < 0.05.

We previously found that drugs that inhibit microglial energy metabolism are a possible mechanism for impairing activation (Abe et al., 2018). Recently, it has been reported that DA inhibits mitochondrial energy metabolism on cultured neuron (Chen et al., 2024). In this study, the effects of DA on cellular metabolism were investigated in primary microglial cells using the seahorse mitochondrial stress test, which assesses oxygen consumption rate (OCR) and extracellular acidification rate (ECAR) (Fig. 6). Metabolic activity is intimately correlated with proinflammatory reactions of microglia, including ROS generation. Here, DA only decreased OCR of maximal respiration at 50 min. However, the flux analysis showed that DA did not significantly impact metabolic activity.

## 4 Discussion

DA has been shown to exert immunosuppressive effects on microglia via D1-like receptors, probably through the elevation of intracellular cAMP levels (Farber et al., 2005, Nishikawa et al., 2023, Tanaka et al., 2024). SKF-81297, a selective D1-like receptor agonist, has been shown to have strong immunosuppressive effects. Other cAMP-elevating agents, such as adrenergic β2 agonists (Ishii et al., 2015, Mori et al., 2002) and rolipram, a cAMP-specific phosphodiesterase inhibitor (Zhang et al., 2002), also suppress LPS-induced proinflammatory activation of microglia. These anti-inflammatory actions may be correlated with the inhibition of the nuclear translocation of NFκB, a proinflammatory transcription factor (Ishii et al., 2015). Moreover, D2-like receptor agonist that suppresses cAMP levels, levodopa also exhibits anti-inflammatory properties in a rat model of Parkinson’s disease (Godinez-Chaparro et al., 2025, Wang et al., 2021). Consequently, the anti-inflammatory and pleiotropic effects of levodopa may not be solely dependent on cAMP signaling. However, levodopa-derived DA has not yet been recognized as a potent immunosuppressant.

This study showed that levodopa acts not only as a DA precursor, but also as an anti-inflammatory agent that suppresses microglial aggravating effects on dopaminergic neurons. If levodopa only had an effect as a DA precursor, the striatal DA level should have decreased after its withdrawal. However, even at 2 weeks after withdrawal, the striatal DA levels of the levodopa-treated rats were higher than those of the vehicle-treated rats. The motor functions of the levodopa-treated rats were improved at 2 weeks after withdrawal. Furthermore, the administration of levodopa prevented pro-inflammatory activation of microglia in the striatum, in accordance with the results that DA prevented LPS-induced pro-inflammatory activation of primary rat microglia. Alternatively, levodopa-derived DA may induce alternative activation of microglia with increased expression of Ym1, Arg1, and bFGF in the ventral midbrains. These levodopa-induced microglial phenotypic changes might contribute the amelioration of the PD model symptoms.

Microglia have long been shown to have detrimental effects on PD pathology (Ayerra et al., 2024, Choudhury et al., 2021a, Choudhury et al., 2011, Ramirez et al., 2017). In response to progressive dopaminergic neuron loss that may cause the generation of damage-associated molecular patterns (DAMPs) (Kumar, 2019), microglia become activated in a proinflammatory manner by recognizing DAMPs, although their pattern recognition receptors, such as Toll-like receptors, are activated. Activated microglia release proinflammatory cytokines (Choudhury et al., 2011, Nishikawa et al., 2023, Tanaka et al., 2024), ROS (Abe et al., 2018), NO (Higaki et al., 2016) and others, all of which are potentially neurotoxic, while aggravating neurodegenerative processes in the SNc. Therefore, many studies have been conducted to determine whether anti-inflammatory drugs can prevent the progressive loss of dopaminergic neurons in both laboratory and clinical settings (Higaki et al., 2016, Yong et al., 2004). Laboratory studies have suggested many drug candidates that prevent neurodegeneration in animal models of PD by suppressing the proinflammatory activation of microglia. However, none of these drugs have been successfully applied in clinical cases (Choudhury et al., 2021a, Wang et al., 2015).

One probable reason for these failures could be that microglia not only have aggravating roles but also have compensatory roles in countering DA loss in the striatum by inhibiting the overactivation of subthalamic neurons in the indirect pathway of the basal ganglia (Aono et al., 2017, Choudhury et al., 2021a). Activated microglia present in the basal ganglia outputs (the substantia nigra pars reticulata and the globus pallidus pars interna) reportedly eliminate glutamatergic receptors from the subthalamic nuclei that are hyperactivated in response to decreased striatal DA levels (Aono et al., 2017). Microglia-mediated synapse elimination ameliorates motor dysfunction. Therefore, immunosuppressive agents may exert both ameliorative effects by suppressing pro-inflammatory microglial activation and detrimental effects by inhibiting beneficial microglial activation in the basal ganglia. Levodopa increases striatal DA levels, leading to the normalization of DA loss-induced hyperactivation of the subthalamic nuclei. Simultaneously, levodopa-derived DA prevented the generation of neurotoxic substances by microglia in the striatum and SNc. Thus, DA, as an immunosuppressant, may not exert conflicting effects on microglia.

Levodopa has been used as a central medicine for PD treatment for decades. If DA has disease-modifying effects as an immunosuppressant that inhibits microglial activation, the modifying effects should have been observed. A clinical study suggests the possibility that levodopa slows the progression of PD and/or has sustained ameliorating effects on PD symptoms (Fahn et al., 2004). However, there are few reports on the disease-modifying effects of levodopa, and the majority of the relevant literature does not support the possibility of disease-modifying effects of levodopa (Verschuur et al., 2019, Vijiaratnam et al., 2021). Thus, the present results obtained through animal experiments are not consistent with the clinical experience. This discrepancy may be caused by the fact that the clinical diagnosis of PD is usually made in the late phase, when most neurons are already degenerated (Bezard et al., 2003, Choudhury et al., 2021a, Zigmond et al., 1984). This may be due to compensatory mechanisms that mask the appearance of motor deficits caused by dopaminergic neuron loss. When most dopaminergic neurons are lost, the characteristics of microglia in the SNc may changes (Ayerra et al., 2024). At this stage, when most neurons degenerate, the anti-inflammatory effects of levodopa may not exert disease-modifying effects. Furthermore, most patients with PD are elderly, and their microglia undergo senescent changes (Choudhury et al., 2021b, Spittau, 2017). In cases where microglia do not undergo senescent changes, levodopa-derived DA may inhibit microglial actions in the basal ganglia outputs to eliminate hyperactive glutamatergic synapses from the subthalamic nuclei (Aono et al., 2017, Choudhury et al., 2021a). The actions of activated microglia in the basal ganglia outputs may be at least one of the mechanisms underlying the compensation for the decrease in striatal DA levels. Levodopa has been shown to reduce the expression of CD68 (Lecours et al., 2020), which is marker for microglial phagocytosis in PD brain (Aono et al., 2017). In sepsis associated encephalopathy (SAE) mice model, levodopa, strongly suppressed neuroinflammation and improved cognitive functions (Li et al., 2020, Nishikawa et al., 2023). Furthermore, levodopa was found to reduce levels of the proinflammatory cytokine IFN-γ in the ischemic brain (Jeon et al., 2024). These results suggest that levodopa could act as a potential immunosuppressant and DA precursor to suppress neuroinflammation in the PD patients’ brains.

## CRediT authorship contribution statement

**Ayane Takenaga:** Data curation. **Junya Tanaka:** Writing – review & editing, Writing – original draft, Investigation, Formal analysis, Conceptualization. **Yuki Nishikawa:** Investigation, Data curation. **Haruto Yamamoto:** Data curation. **Masahiro Nagai:** Writing – review & editing, Writing – original draft, Supervision. **Naoki Abe:** Investigation, Data curation. **Tasuku Nishihara:** Writing – review & editing, Writing – original draft. **Noriyuki Miyaue:** Writing – review & editing, Writing – original draft, Data curation, Conceptualization. **Ikuko Takeda:** Data curation, Conceptualization. **Choudhury Mohammed E:** Writing – review & editing, Writing – original draft, Visualization, Validation, Supervision, Software, Resources, Project administration, Methodology, Investigation, Funding acquisition, Formal analysis, Data curation, Conceptualization.

## Ethics approval and consent to participate

Not applicable.

## Consent for publication

Not applicable.

## Funding

This work was supported by grants from the 10.13039/501100008665Osaka Medical Research Foundation for Intractable Diseases [28–2–22 (MEC)], and from the Grant-in-Aid for Scientific Research (C) to [24K12069 (MEC), 23K08383 (TN), and 23K08333 (NA)], and the Grant-in-Aid for Early-Career Scientists to [21K15699 (NM) from the Japan Society for the Promotion of Science (JSPS).

## Declaration of Competing Interest

The authors declare that they have no known competing financial interests that could have appeared to influence the work reported in this paper.

## Data Availability

The datasets used and/or analyzed in the present study are available on reasonable request.
