# Supplementary material for: Levodopa exerts neuroprotective effects by suppressing microglial proinflammatory activation in a rat hemi-Parkinson’s disease model
Source: IBRO Neurosci Rep. 2025 Dec 4;19:1053–62. doi: 10.1016/j.ibneur.2025.12.001 (PMC12753262; doi:10.1016/j.ibneur.2025.12.001)
Supplement: Supplementary file 1 — Supplementary material [file mmc1.pdf]

Levodopa exerts neuroprotective effects by suppressing microglial proinflammatory activation in a rat hemi-Parkinson's disease model

Noriyuki Miyaue[1, *], Mohammed E. Choudhury[2,3 *, †], Ikuko Takeda[4, 5], Junya Tanaka[2, 3], Ayane Takenaga[1], Haruto Yamamoto[1], Yuki Nishikawa[2], Naoki Abe[2], Masahiro Nagai[1] and Tasuku Nishihara[2].

Author details

1 Department of Clinical Pharmacology and Therapeutics, Ehime University Graduate School of Medicine, Toon, Ehime, 791-0295, Japan

2 Department of Anesthesia and Perioperative Medicine, Ehime University Graduate School of Medicine, Toon, Ehime, 791-0295, Japan

3 Department of Molecular and Cellular Physiology, Ehime University Graduate School of Medicine, Toon, Ehime, 791-0295, Japan

4 Department of Anatomy and Molecular Cell Biology, Nagoya University Graduate School of Medicine, Nagoya, Aichi, 466-8550, Japan.

5 Division of Multicellular Circuit Dynamics, National Institute for Physiological Sciences, Okazaki, Aichi 444-8585, Japan

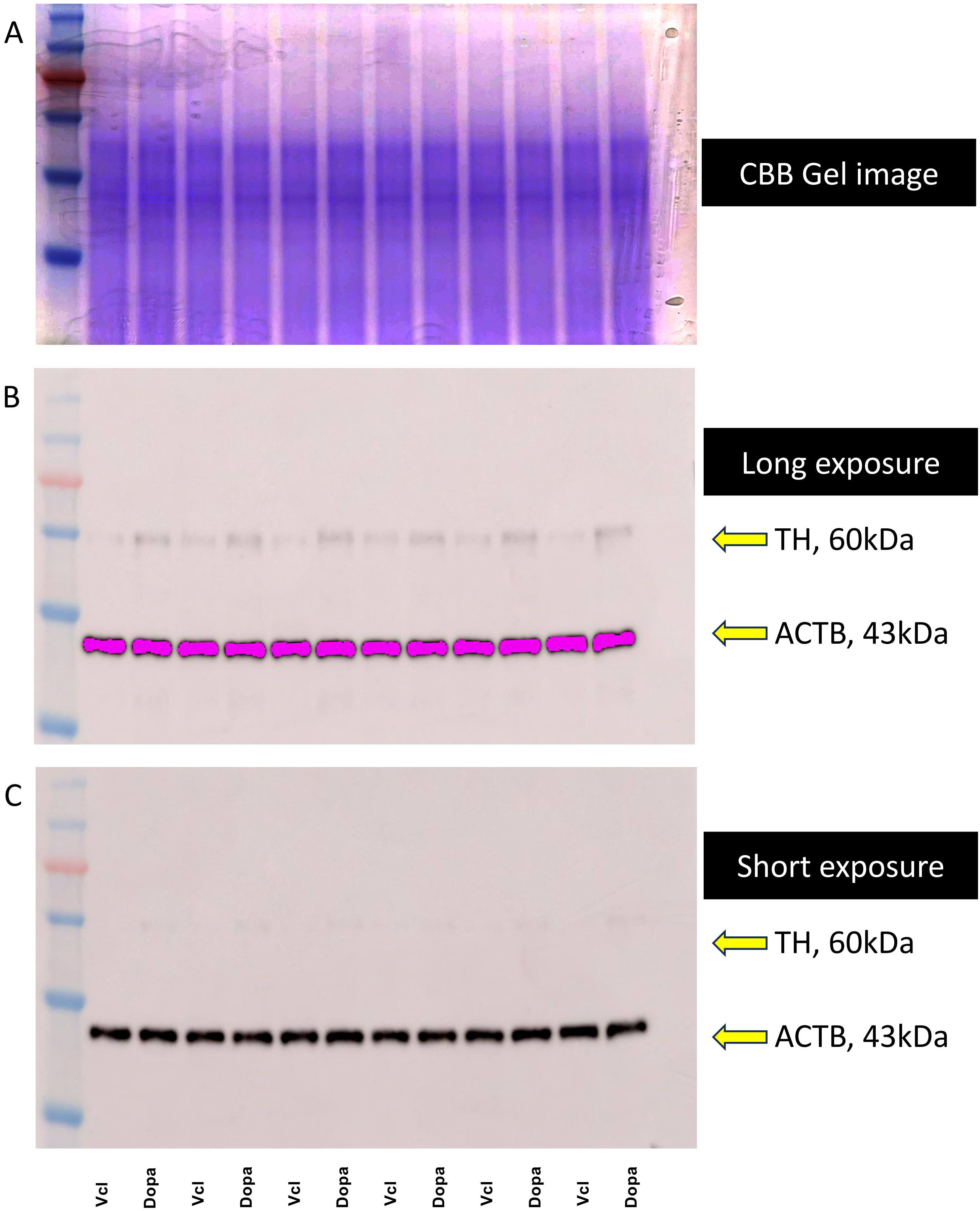

Supplementary Figure: Ameliorative effects of levodopa on PD model rats: Gel and membrane images of immunoblot showing TH and ACTB immunoreactivity in the ventral midbrain of vehicle- and levodopa-treated rats (n=6).
