# Supplementary material for: Levodopa exerts neuroprotective effects by suppressing microglial proinflammatory activation in a rat hemi-Parkinson’s disease model
Source: IBRO Neurosci Rep. 2025 Dec 4;19:1053–62. doi: 10.1016/j.ibneur.2025.12.001 (PMC12753262; doi:10.1016/j.ibneur.2025.12.001)
Supplement: Supplementary file 2 — Supplementary material [file mmc2.pdf]

Supplementary Table : Primers for qPCR

Name		Sequence
Arg1	Fwd	GGAACGAAACGGGAAGGTAA
	Rvs	CTGTTCGGTTTGCTGTGATG
bFGF	Fwd	GACCCACACGTCAAACTACA
	Rvs	GACTCCAGGCGTTCAAAGAA
Cd32	Fwd	GCTTTCTGCCTAGTGATGTGC
	Rvs	CATTTGTCCTGTGGAGCCTT
Cd206	Fwd	TGCTTGTTGATTGCCACTTTC
	Rvs	GCAGAGTTGACATGAGACCTAC
D1r	Fwd	GAGTGGTTGGGGGAAGTCTG
	Rvs	GAGAAATCCCTCTCCGCTGG
D2r	Fwd	TCCTGAACCTGTGTGCCATC
	Rvs	GGACAGGACCCAGACAATGG
D3r	Fwd	CTTCTACGTTCCCTTCGGGG
	Rvs	ACTGGCTGTTCTGTCGAGTG
D4r	Fwd	CAGACACCCACCAACTAC
	Rvs	TTGAAGATGGAGGCGGTG
D5r	Fwd	CTCCTTCATCCCGGTCCAAC
	Rvs	TCCGTCCTCCCTTCTAGCTC
Gapdh	Fwd	GAGACAGCCGCATCTTCTTG
	Rvs	TGACTGTGCCGTTGAACTTG
iNOS	Fwd	AGGGAGTGTTGTTCCAGGTG
	Rvs	TCCTCAACCTGCTCCTCACT
Th	Fwd	TGTGTCCGAGAGCTTCAATG
	Rvs	GGGCTGTCCAGTACGTCAAT
Ym1	Fwd	AAGGTGACCCTTGACAGTGG
	Rvs	TCCCAGCCTTAGCATGTACC
